# Comprehensive strategies in breast cancer-related lymphedema prevention: insights from a multifaceted program

**DOI:** 10.3389/fonc.2024.1418610

**Published:** 2024-07-16

**Authors:** Fardeen Bhimani, Maureen McEvoy, Yu Chen, Anjuli Gupta, Jessica Pastoriza, Arianna Cavalli, Liane Obaid, Carolyn Rachofsky, Shani Fruchter, Sheldon Feldman

**Affiliations:** ^1^ Breast Surgery Division, Department of Surgery, Montefiore Medical Center, Montefiore Einstein Comprehensive Cancer Center, Bronx, NY, United States; ^2^ Albert Einstein College of Medicine, Bronx, NY, United States

**Keywords:** breast cancer-related lymphedema, BCRL, SOZO®, bioimpedance spectroscopy, BIS, lymphedema education, axillary reverse mapping, ARM

## Abstract

**Background:**

Breast cancer-related lymphedema (BCRL) profoundly impacts patients’ quality of life, causing heightened depression, anxiety, and physical limitations. Surgical removal of the axillary nodes, combined with radiation therapy, is a significant risk factor for BCRL. Smarter axillary surgery, coupled with early detection and fostering lymphedema education, significantly improves BCRL management, promoting timely diagnosis and treatment. A lymphedema prevention program encompassing all these factors can significantly aid in preventing, treating, and reducing the severity of BCRL cases. Therefore, our study aims to share our insights and experiences gained from implementing a lymphedema prevention program at our institution.

**Methods & Results:**

At our institution, axillary reverse mapping (ARM) is performed on all patients undergoing axillary surgery. We surveil these patients with pre- and postoperative SOZO^®^ measurements using bioimpedance spectroscopy to detect sub-clinical lymphedema. Concerning education, we use a 3-pronged approach with surgeons, nurse practitioners, and video representation for patients. We have had 212 patients undergo the ARM procedure since 2019, with three (1.41%) developing persistent lymphedema.

**Conclusion:**

Our study underscores the significance of a comprehensive lymphedema prevention program, integrating smarter axillary surgery, early detection, and patient education. The lymphedema rate of 1.41% not only validates the success rate of these interventions but also advocates for their widespread adoption to enhance the holistic care of breast cancer survivors. As we continue to refine and expand our program, further research, and long-term follow-up are crucial to improve prevention strategies continually and enhance the overall well-being of individuals at risk of BCRL.

## 1 Background

Breast cancer-related lymphedema (BCRL) is a common complication associated with breast cancer treatment (1). It occurs as a result of disruptions in the lymphatic system, impeding proper drainage from lymphatic vessels and leading to the accumulation of protein-rich lymph fluid in the interstitial space (2, 3). This surplus fluid can result in abnormal swelling on the treated side’s breast, trunk, or upper extremity. The development of BCRL is multifaceted, stemming from a combination of treatment-related factors such as surgical interventions, radiation therapy, and chemotherapy (4–10). In addition to these treatment-related factors, patient-specific factors also play a key role in BCRL development. These include advanced age, race, an elevated body mass index (BMI), cellulitis, and variations in limb volume (11–14). Depending upon the severity of edema, BCRL symptoms include arm tightness, tingling, numbness, heaviness/fullness, pain, and impaired limb function (2, 15, 16). As fluid accumulation progresses, it may culminate in fibrosis, causing further dexterity issues (17). Apart from physical symptoms, BCRL also affects a patient’s quality of life (QoL); prior studies have demonstrated that BCRL significantly diminishes a patient’s QoL, leading to higher incidences of depression and anxiety, as well as greater physical impairment such as the risk of developing cellulitis and angiosarcoma when compared to individuals without BCRL (18–22). Therefore, identification and intervention for BCRL is of paramount importance, as it exerts a profound impact on the patient’s overall well-being.

A lymphedema prevention program could significantly aid in preventing, treating, and reducing the severity of BCRL cases. An important cause for the development of BCRL is the removal of arm nodes that drain the upper extremity at the time of surgery (23, 24). Adopting smarter axillary surgery, such as Axillary Reverse Mapping (ARM), can potentially reduce lymphedema by identifying and maintaining upper lymphatic drainage during Axillary Lymph Node Dissection (ALND) and Sentinel Lymph Node Biopsy (SLNB) (25). Several studies have attested to the effectiveness of ARM in diminishing the prevalence of lymphedema (26–31). Additionally, early detection also plays a crucial role in the management of BCRL. Multiple diagnostic modalities exist for the detection of BCRL, from traditional tape measurement to sophisticated techniques like Perometry and Bioimpedance Spectroscopy (BIS) (32–34). Moreover, fostering lymphedema education and awareness can significantly contribute to timely diagnosis and treatment. Prior studies have underscored the lack of knowledge and awareness among women diagnosed with breast cancer regarding lymphedema (16, 35). Therefore, our study aims to share our insights and experiences gained from implementing a lymphedema prevention program at our institution. Our multifaceted approach encompasses smarter axillary surgery, early detection modality, and a commitment to educating and raising awareness, collectively contributing to a more proactive stance against BCRL.

## 2 Smarter axillary surgery

### 2.1 Definition and significance

Smarter axillary surgery refers to novel surgical techniques and approaches to lower the risk of complications, particularly lymphedema, associated with procedures like ALND and SLNB. The surgical techniques aim to preserve lymphatic drainage pathways during surgery to minimize lymphatic system disruption. These include but are not limited to ARM, lymphatic microsurgical preventive healing approach (LyMPHA), simplified lymphatic microsurgical preventing healing approach (S-LyMPHA), and lymphatic re-approximation. Smarter axillary surgeries can be extremely beneficial in patients where ALND cannot be avoided by decreasing the level of morbidity, especially when 82% of women have at least one upper extremity symptom following ALND (36).

### 2.2 Types of smarter axillary surgeries

We are increasingly avoiding axillary surgery for many patients who are elderly and clinically node negative per SSO *Choosing Wisely* guidelines and the recently published SOUND trial result. For those patients deemed to benefit from axillary surgery, we routinely perform the ARM technique (37, 38). The ARM technique can distinguish between lymphatic drainage pathways of the breast and the arm using agents like blue dye, ICG-Indocyanine green fluorescence, or radioisotope, which allow visualization of lymphatic channels in the upper extremities. The technique involves the injection of the agent dermally and subcutaneously in the upper inner arm along the medial intramuscular groove of the ipsilateral arm to locate the draining lymphatics from the arm and sparing the nodes identified by the agent to prevent lymphedema (39). Multiple studies have been conducted highlighting the effectiveness of the technique in reducing BCRL (26–31).

The LyMPHA procedure is usually performed in patients when a malignant node is identified, draining the arm. In such instances, a lymphovascular anastomosis is performed to proactively prevent secondary lymphedema. Plastic surgeons usually perform the procedure during cancer surgery and require a microscope. Following the excision of the blue arm node, the blue lymphatic vessel is placed inside a vein branch with a competent valve and secured with an 8-0 permanent suture (40).

The S-LyMPHA is a both simplified and modified technique that an operating surgeon usually performs without a microscope. During ALND, the transected blue lymphatic channels are identified. Upon completion of the dissection, these channels are meticulously dissected and invaginated into the cut end of a neighboring vein using a sleeve technique, secured with two 7-0 nonabsorbable sutures.

Lymphatic re-approximation involves the reanastomosis of afferent and efferent lymphatics. Following the removal of the Arm nodes (which drains both the breast and axilla), transected lymphatics can undergo re-approximation where afferent and efferent fatty bundles are reapproximated with 3-0 vicryl.

Notably, not all ALND patients undergo lymphatic re-approximation/LyMPHA procedure. In certain cases, a selective ALND is performed to preserve arm nodes that are not grossly malignant. However, if the arm nodes are deemed malignant, ALND with LYMPHA is performed, which involves removing the affected nodes as part of the ALND procedure and then restoring lymphatic drainage via LyMPHA.

### 2.3 Implementation in our program

At our institution, we perform the ARM technique on all patients undergoing SLNB and ALND. To perform the ARM technique, we primarily use a blue dye 15 minutes before making an incision in the axilla. The dye is injected in the upper medial aspect of the arm and massaged for approximately 5 minutes. For patients undergoing simultaneous reconstruction procedures alongside cancer surgery, we have implemented the LyMPHA procedure. In cases where a malignant blue node is removed, the breast surgeons routinely perform a lymphatic re-approximation procedure in the setting of SLNB. This additional step, similar to the LyMPHA procedure, has been highly effective in contributing to lower rates of lymphedema.

## 3 Early detection

### 3.1 Importance of early detection

According to the ISL, lymphedema is categorized into 4 stages (41). Stage 0 is defined as a subclinical stage where the patient has an increase in limb volume but develops no signs and symptoms. Stage 1 manifests as early edema that shows improvement with limb elevation. Stage 2 is characterized by pitting edema that persists even with elevation. Stage 3 involves fibroadipose deposition and skin changes. Notably, Stages 0 and 1 are reversible and treatable in the majority of cases, whereas Stages 2 and 3 are deemed irreversible (42). Early detection and treatment are crucial for Stages 0 and 1 to prevent the patient from experiencing a lifetime of morbidity, highlighting the significance of early detection.

### 3.2 Diagnostic modalities

Various modalities can be employed to diagnose BCRL, encompassing basic physical examination using a measuring tape, limb immersion technique, Perometry, BIS, and Lymphoscintigraphy (43). However, each modality has its distinct strengths and limitations. Tape measurements are reliable, validated, and cost-effective, yet necessitate strict protocols and training (44). The water displacement technique with limb immersion is used for volumetric assessment but is time-consuming, involves bulky equipment, requires specific hospital protocols, and is discouraged in the presence of skin lesions. Moreover, it has the potential for inter- and intra-observer variations (45–50). Perometry is a validated and reliable tool capable of detecting subclinical lymphedema, but its cost, lack of portability, and need for dedicated space limit accessibility (43, 51–53). BIS is efficient, accurate, and aids in early detection of BCRL (54–56). However, it comes with monthly software and data management fees (43). Lymphoscintigraphy is considered the gold standard in diagnosing BCRL, offering direct visualization of lymphatic function (57). A radiotracer injected into the hand or wrist is absorbed by lymphatic vessels and nodes during this procedure. A single-photon emission computed tomography is used to evaluate dermal backflow and identify lymphatic blockages. While Lymphoscintigraphy is diagnostically accurate, it’s an invasive procedure, demands skilled personnel, and is costly (43).

### 3.3 Utilization in our program

**Figure 1:**
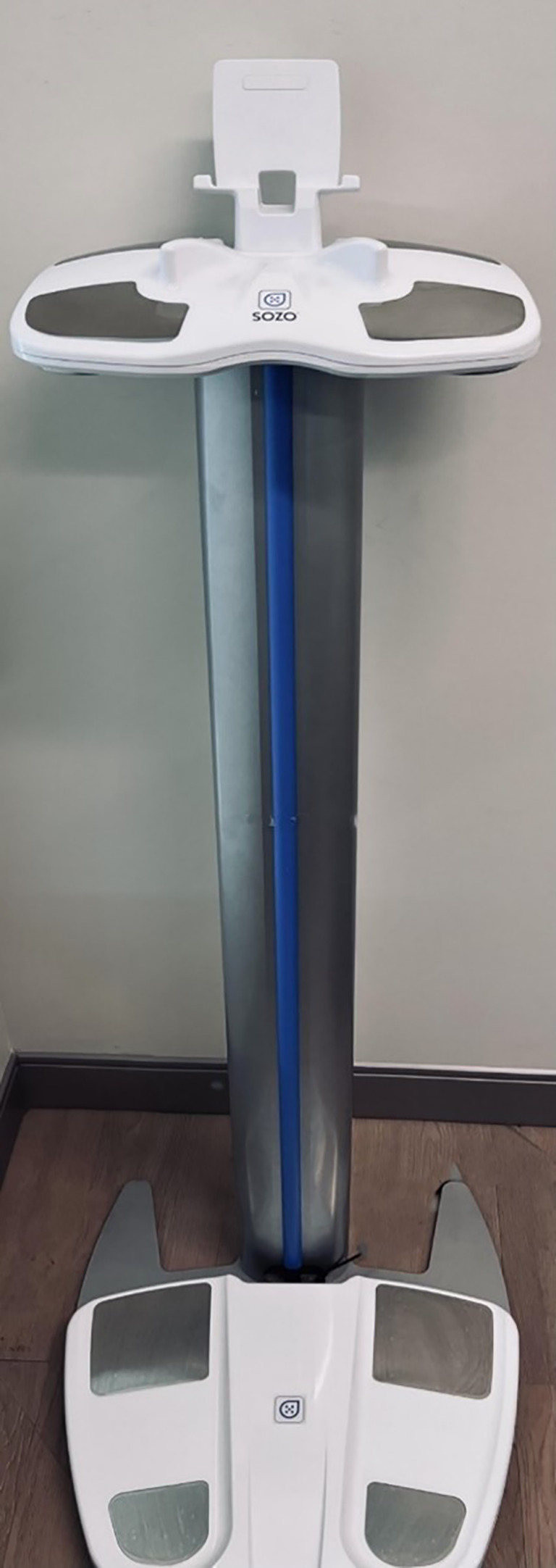
SOZO^®^ Device.

We utilize the SOZO^®^ device, which uses BIS to assess tissue resistance to an electrical current and converts it into a score in real-time reflecting interstitial fluid content (58) (**Figure 1**). Detecting sub-clinical lymphedema enables early intervention in BCRL, leading to timely resolution (32, 51, 59). Patients undergoing SLNB and/or ALND receive a preoperative L-Dex score assessment, followed by postoperative monitoring at 3 to 6-month intervals to detect sub-clinical lymphedema. Sub-clinical lymphedema is defined as an absolute L-Dex score exceeding +10 and an increase of 6.5 or more from baseline. SOZO^®^ scores at each follow-up are compared to preoperative baseline scores to monitor changes. This has enabled prompt initiation of interventions like prescribing compression garments or physical therapy, leading to resolution for many patients and highlighting its clinical significance.

We also consider subjective factors when evaluating arm symptoms related to lymphedema. We use the QuickDASH questionnaire, a validated patient-reported outcome tool that strongly correlates with the original DASH and has been used to assess the effectiveness of lymphedema treatment (60, 61). QuickDASH introduces an important subjective dimension to our safety measures. We administer this questionnaire preoperatively to establish a baseline and at each follow-up.

## 4 Patient education

### 4.1 Importance of patient education

Lymphedema education and awareness could significantly contribute to early diagnosis and treatment. Studies conducted by Fu et al. (16, 35) have revealed that lack of awareness about lymphedema prevents women from seeking necessary support and assistance, thereby exacerbating their suffering and distress. Furthermore, Thomas-MacLean et al. (60) highlighted a lack of knowledge about BCRL among healthcare providers across various disciplines, encompassing surgeons, oncology department staff, and family physicians. Thus, physicians must remain vigilant, recognizing BCRL and its symptoms while prioritizing comprehensive patient education.

### 4.2 Our educational approach

Our comprehensive patient education initiatives aim to fill knowledge gaps and empower patients through a three-pronged approach. The surgeon leads preoperative discussions on lymphedema, emphasizing the risks and benefits of lymph node surgery, including its causes, signs, symptoms, associated risks, and available treatment options. Subsequently, nurse practitioners offer detailed oral and written explanations, supplemented by educational materials, providing patients with comprehensive insights into their postoperative care. Providing written and oral information contributes to better outcomes, as patients who receive guidance exhibit improved management of BCRL (61). Furthermore, before their SOZO^®^ measurements, patients view an informative graphic video available in both English and Spanish, illustrating the workings of the lymphatic system, the removal of nodes, stages of lymphedema, associated symptoms, and the measurement process to provide a visual representation for better understanding (See **Supplementary Material** for video link). With the introduction of our educational approach, we’ve observed a positive shift in patient engagement and proactivity. During follow-ups, patients are now actively requesting their SOZO^®^ measurements without prompting from providers. Additionally, patients are independently researching lymphedema prevention online and incorporating lymphedema exercises into their routines, showcasing an increased awareness and proactive approach to their well-being.

## 5 Results

We retrospectively analyzed data from 2019 to 2022, during which our institution performed the ARM procedure on 212 patients. The mean age and BMI, along with standard deviation, accounted for 57.4 + 11.3 years and 29.5 + 5.7 kg/m^2^, respectively. A majority of our patients (83%) belonged to ethnic minorities. Of the 212, 143 (67.5%) underwent lumpectomy, and 69 (32.5%) had mastectomy. Additionally, 177 (83.5%) had SLNB, and 35 (16.5%) underwent ALND (**Table 1**).

**Table 1:** Summary of patient data.

**Total Patients**	212
**Age, mean (SD)**	57.4 (11.3)
Race/Ethnicity	
Asian	8 (3.8%)
African American	72 (34%)
White	23 (10.8%)
Hispanic	96 (45.3%)
Unknown	13 (6.1%)
**BMI, mean (SD)**	29.5 (5.7)
Laterality	
Left	98 (46.2%)
Right	114 (53.8%)
Pathology	
DCIS IDC	9 (4.2%) 174 (82.1%)
DCIS, IDC	3 (1.4%)
ILC/Mixed Pathology	26 (12.3%)
Stages	
0	9 (4.2%)
1	115 (54.2%)
2	56 (26.4%)
3	16 (7.5%)
4	5 (2.4%)
Unknown	11 (5.2%)
Lumpectomy	143 (67.5%)
Mastectomy	69 (32.5%)
SLNB	176 (83%)
Target Axillary Dissection	7 (4%)
ANLD	36 (17%)
Initial SLNB Treatment	24 (68.6%)
Positive Nodes (Total)	67
1 – 4	55 (82.1%)
5 – 9	8 (11.9%)
10 +	4 (6%)
**Blue Nodes Identified** SLNB ALND	47 (22.2%) 36 (76.6%) 11 (23.4%)
**Blue Nodes Excised** SLNB ALND	33 (70.2%) 24 (72.7%) 9 (27.3%)
**Crossover Nodes** SLNB ALND	17 (53.1%) 15 (88.2%) 2 (11.8%)
**Number of Crossover Nodes that were Positive** SLNB ALND	7 (41.2%) 4 (57.1%) 3 (42.9%)
**Blue Lymphatics Visualized** SLNB ALND	55 (25.9%) 39 (70.9%) 16 (29.1%)
**Patient(s) with Persistent Lymphedema** SLNB ALND	3 (1.4%) 0 3 (100%)
**Patient(s) with Transient Lymphedema** SLND ALND	15 (7%) 12 (80%) 3 (20%)
**Lymphatic Re-approximation**	15 (7.1%)
SNLB	13 (87%)
ALND	2 (13%)
**Blue Lymphatics observed**	15 (100%)
**Number of Blue Nodes Identified**	12 (80%)
**Number of Crossover Nodes Removed**	5

### 5.1 ARM procedure

ARM was performed on all 212 patients undergoing axillary surgery of which 176 (83%) patients underwent SLNB, and 36 (17%) underwent ALND. Intraoperatively, blue nodes were identified in 47 patients (22.2%), 36 during SLNB, and 11 during ALND. Blue lymphatics were observed in 55 patients (23.4%), with 39 identified during SLNB and 16 during ALND. Of the identified blue nodes, 68.1% (n=32) were excised, and 53.1% (n=17) were diagnosed as crossover nodes, which are defined as sentinel nodes that are blue. Among the 67 patients with positive nodes, 19 had blue nodes removed, and 7 were crossover nodes. One patient experienced persistent lymphedema after crossover node removal.

### 5.2 Lymphatic re-approximation

In our cohort, lymphatic re-approximation was conducted on 15 patients, with 13 undergoing SLNB and 2 undergoing ALND. Among these patients, 12 had blue nodes identified, including 5 crossover nodes that were subsequently removed. Blue lymphatics were observed in all 15 patients. Notably, one patient undergoing Lymphatic re-approximation developed persistent lymphedema.

### 5.3 Lymphedema rate

Lymphedema assessment using SOZO^®^ involved a comparison of pre-operative and post-operative L-Dex scores. The mean pre-operative L-Dex score for the entire cohort was 0.12 ± 6.4 (normal range -10 to +10). Regarding BCRL, 18 patients exhibited an elevated L-Dex score during post-operative follow-up. Notably, 15 of these patients saw a resolution of lymphedema, as indicated by a decrease in their L-Dex score at the 3-month follow-up. Consequently, our overall lymphedema rate was 1.41% (3/212), with rates of 0% (0/176) following SLNB and 8.3% (3/36) after ALND. The remaining three patients who developed BCRL had all undergone ALND; two of them continued to have persistent lymphedema after blue node removal, with L-Dex scores of 23.77 and 22.39, respectively, at the 2-year follow-up. The third patient died from metastatic cancer progression, and her final L-Dex score was 77.8. All individuals with persistent lymphedema underwent treatment comprising a combination of compression sleeve therapy and physical therapy. Among the 15 individuals with transient lymphedema, 9 were treated with a sleeve and/or physical therapy, while the remaining 6 experienced spontaneous resolution of their condition during follow-up.

## 6 Discussion

Our multifaceted lymphedema prevention program has significantly reduced our lymphedema rate through smarter axillary surgery and early detection using SOZO^®^. As a result, our lymphedema rate is significantly lower than previously reported data (14, 62, 63). Most patients in our cohort belong to ethnic minorities and had a higher BMI. This is particularly noteworthy, as prior studies have illustrated a two-fold increased risk of lymphedema in ethnic minority populations, particularly African-Americans (14, 62, 63). Flores et al. (14) reported a rate of lymphedema of 40.4% (42/104) among African-American patients, characterized by signs and symptoms. In contrast, our study found a significant reduction in the incidence of BCRL, particularly among African-American patients (3/72) (Chi-square test p<0.001). Moreover, having a BMI exceeding 25 kg/m² is a significant risk factor for developing lymphedema (64, 65). According to a study by Meeske et al. (66), women with a BMI greater than 25 kg/m² experienced a twofold increase in arm lymphedema, while those with a BMI exceeding 30 kg/m² had a threefold increase. Notably, despite our study participants having a mean BMI of 29.5 ± 5.7 kg/m², our observed rate of lymphedema was significantly lower. Our overall lymphedema rate of 1.41% is similar to that documented by Tummel et al. (67), who conducted a prospective single-arm phase II trial to determine if ARM prevents lymphedema and found that objective lymphedema rates for SLNB and ALND were 0.8% and 6.5%, respectively. Similarly, Yue et al. (31) randomized 265 patients to undergo ALND or ALND+ARM and reported that lymphedema occurred in 33% of the ALND and 6% of the ALND+ARM patients. Likewise, Yuan et al. (26) and Faisal et al. (27) performed ARM in ALND patients and found a lymphedema rate of 3.3% and 4.2% following ARM compared to 15.3% and 16.7% after ALND, respectively.

Furthermore, the LyMPHA and S-LyMPHA techniques have also shown effectiveness in lowering the incidence of BCRL. A study by Boccardo et al. (68) highlighted that ALND patients undergoing LyMPHA experienced a significantly lower lymphedema incidence of 4.34% compared to 30.43% in those without LyMPHA at their 6-month follow-up. In a subsequent study, they observed a 4% lymphedema rate after ALND over a 4-year follow-up period in 74 patients (69). Similarly, Feldman et al. (70) demonstrated that the lymphedema rate was 3 (12.5%) out of 24 patients who underwent a successfully completed LyMPHA procedure, compared to 50% among patients in whom LyMPHA was unsuccessful. Likewise, Ozmen et al. (71) found that patients undergoing S-LyMPHA had a lower rate of 3%, compared to 19% in those without S-LyMPHA (p=0.001). Apart from these, the lymphatic-reapproximation technique, when incorporated alongside the ARM procedure at our institution, demonstrated a low lymphedema incidence of only 1 out of 15 patients. Tummel et al. (67) similarly incorporated this technique into their ARM procedure, revealing a statistically significant difference in lymphedema outcomes. Patients undergoing re-approximation experienced a 0% rate, whereas those without re-approximation exhibited a BCRL rate of 18.7% (p=0.009) (67).

The integration of SOZO^®^ into our program has been a transformative asset, significantly enhancing our ability to detect sub-clinical lymphedema. At our institution, 18 patients were diagnosed as having BCRL via SOZO^®^. However, the prompt treatment led to a resolution in 15 of these patients, highlighting the role of SOZO^®^ in diagnosing early-stage BCRL and preventing these individuals from enduring lifelong morbidity. The PREVENT trial underscored the precision of BIS in identifying patients who would benefit from early compression treatment compared to traditional tape measurements (72). Similarly, in a prospective study, high-risk BCRL patients undergoing ALND had regular BIS assessments every 3-6 months. Those with sub-clinical lymphedema received physical therapy, compression garments, and lymphedema education. Notably, 4.4% developed clinically evident BCRL at the 19-month follow-up (54). This emphasizes the importance of establishing basic surveillance models to triage individuals for timely preventive measures effectively. However, it is crucial to acknowledge that accurate SOZO^®^ measurements may necessitate basic training and require a dedicated space and a monthly subscription. Notably, Current Procedural Terminology (CPT^®^) codes facilitate reimbursement for SOZO^®^ measurements, rendering the surveillance program financially feasible, thereby reducing both lymphedema incidence and overall financial burden. We adopted the QuickDASH questionnaire in 2022. The data is premature to draw conclusions. Nevertheless, the authors strongly advocate implementing a robust lymphedema prevention program. Such initiatives are pivotal in substantially reducing the incidence of BCRL.

## 7 Conclusion

Our study underscores the significance of a comprehensive lymphedema prevention program, integrating smarter axillary surgery, early detection, and patient education. The lymphedema rate of 1.41% not only validates the success rate of these interventions but also advocates for their widespread adoption to enhance the holistic care of breast cancer survivors. As we continue to refine and expand our program, further research, and long-term follow-up are crucial to continually improve prevention strategies and enhance the overall well-being of individuals at risk of BCRL.

## Data availability statement

The raw data supporting the conclusions of this article will be made available by the authors, without undue reservation.

## Ethics statement

The studies involving humans were approved by Office of Human Research Affairs - Albert Einstein College of Medicine/Montefiore Medical Center. The studies were conducted in accordance with the local legislation and institutional requirements. The ethics committee/institutional review board waived the requirement of written informed consent for participation from the participants or the participants’ legal guardians/next of kin because of the retrospective design of the study.

## Author contributions

FB: Conceptualization, Data curation, Formal analysis, Investigation, Project administration, Writing – original draft, Writing – review & editing. MM: Project administration, Visualization, Writing – original draft, Writing – review & editing. YC: Data curation, Formal analysis, Resources, Software, Visualization, Writing – original draft, Writing – review & editing. AG: Project administration, Visualization, Writing – original draft, Writing – review & editing. JP: Project administration, Supervision, Writing – original draft, Writing – review & editing. AC: Data curation, Investigation, Writing – original draft, Writing – review & editing. LO: Data curation, Investigation, Writing – original draft, Writing – review & editing. CR: Data curation, Investigation, Writing – original draft, Writing – review & editing. SFr: Investigation, Methodology, Writing – original draft, Writing – review & editing. SFe: Conceptualization, Methodology, Project administration, Supervision, Validation, Writing – original draft, Writing – review & editing.
