# Supplementary material for: Comprehensive strategies in breast cancer-related lymphedema prevention: insights from a multifaceted program
Source: Front Oncol. 2024 Jul 16;14:1418610. doi: 10.3389/fonc.2024.1418610 (PMC11286467; doi:10.3389/fonc.2024.1418610)
Supplement: Supplementary file 1 [file DataSheet_1.docx]

**Comprehensive Strategies in Breast Cancer-Related Lymphedema Prevention: Insights from a Multifaceted Program**

**Supplemental File: Lymphedema Education Video**

English: [Lymphedema Educational Video.mp4](https://montefioreorg-my.sharepoint.com/:v:/g/personal/fbhimani_montefiore_org/EUHFwnENQD9LvfjFmU5ADZkBenHMR1Dj9lHy8UjPVe7DwQ?e=ndEGNZ)

Spanish: [Lymphedema Spanish Video Final.mp4](https://montefioreorg-my.sharepoint.com/:v:/g/personal/fbhimani_montefiore_org/EbABqyksXJFAvJw1Bo_cZKsBR4-NSCevxguiPrQ6r56IZQ?nav=eyJyZWZlcnJhbEluZm8iOnsicmVmZXJyYWxBcHAiOiJTdHJlYW1XZWJBcHAiLCJyZWZlcnJhbFZpZXciOiJTaGFyZURpYWxvZy1MaW5rIiwicmVmZXJyYWxBcHBQbGF0Zm9ybSI6IldlYiIsInJlZmVycmFsTW9kZSI6InZpZXcifX0%3D&e=c7ONxP)
